# A Flexible, Self‐Floating Composite for Efficient Water Evaporation

**DOI:** 10.1002/gch2.201800085

**Published:** 2018-11-04

**Authors:** Zhenxing Fang, Shihui Jiao, Boran Wang, Wen Yin, Guangsheng Pang

**Affiliations:** ^1^ State Key Laboratory of Inorganic Synthesis and Preparative Chemistry College of Chemistry Jilin University Changchun 130012 P. R. China; ^2^ College of Science and Technology Ningbo University Ningbo 315200 P. R. China

**Keywords:** desalination, composites, carbon, water scarcity, solar energy

## Abstract

A flexible, self‐floating W_18_O_49_/carbon foam composite is fabricated by calcining melamine foam with W_18_O_49_ as an adsorbate in N_2_ atmosphere. This self‐floating property is simply realized by a carbonization process other than the complicated surface modification process. The simple synthesis procedure helps to increase not only the solar absorption but also the retention of W_18_O_49_ in the porous net structure. This composite absorbs almost the whole solar spectrum and generates localized heat at the surface, which is beneficial for water evaporation. Its water evaporation rate is 6.6 times higher than that of pure water. It has a stable cyclic performance over ten cycles under the illumination of simulated sunlight (500 W Xe lamp). Its flexibility makes it easy to reuse and transfer, which is evaluated by the bending deformation test. The W_18_O_49_/carbon foam composite is a prospective material in solar energy conversion field, and the preparation procedure is feasible to scale‐up.

## 1 Introduction

Water scarcity has become one of the most serious global problems due to the increasing demands and unreasonable waste of water. Seawater desalination is a very promising means to gain fresh water.^1^ Osmosis membrane technology,^2^, ^3^, ^4^, ^5^ solar desalination technology,^6^, ^7^, ^8^, ^9^ and some other technologies^10^, ^11^ have been used for desalination. Among them, efficient water evaporation technique has attracted tremendous attention due to its distinct advantages, such as inexhaustibility, safety, and environmental friendliness. However, this technique suffers from low efficiency due to the heating of bulk water which is unnecessary for evaporation.^12^ And this challenge could be solved by utilizing the self‐floating devices which generates localized heat at the interface of water and air.^13^, ^14^, ^15^

To enable efficient solar desalination, the broadband and efficient light absorption of this self‐floating composite is the primary condition.^8^, ^9^ The first reported material used for solar steam generation is gold nanoparticle which could engender heat under laser illumination because of its distinct surface plasmon resonance effect.^16^ However, there are some main disadvantages which hinder its industrial application^17^: i) the high cost for scale‐up production of noble metal–based film, ii) these films are fragile and hard to transfer, which make them poorly durable, iii) heat loss occurs by thermal diffusion to the nonevaporative portion of water due to its thin thickness. With the development of nanomaterial science and nanotechnology, many other solar thermal conversion materials are exploited such as carbon‐based materials and semiconductors with lots of free carriers.^18^, ^19^, ^20^, ^21^, ^22^, ^23^, ^24^, ^25^ Whereas the preparation procedure of carbon‐based materials such as graphene and carbon nanotube is complex and hard to control, which is not suitable for large scale production.^26^ Thus, the feasible and controllable synthesis of semiconductors such as titanium oxide (Ti_2_O_3_),^19^ copper sulfide (Cu_2−_ *_x_*S),^22^, ^23^ tungsten oxide (WO_3−_ *_x_*)^24^, ^25^ make them the most promising alternatives for noble metals. The other condition for improving evaporation efficiency is the structure design of the material. A lot of bio‐inspired works have revealed that porous design can effectively enhance the solar light scattering inside the pores to increase the light absorption.^27^, ^28^, ^29^, ^30^, ^31^, ^32^, ^33^ Furthermore, it can focus the generated heat in a limited region to reduce the heat loss which is caused by thermal diffusion to bulk water.

Melamine foam is an optimal substrate due to its high porosity, low density, and hydrophilicity. Whereas, melamine foam will sink after absorbing water and this could be tuned by carbonization process (annealing in inert atmosphere). Thus, we choose the porous melamine foam as the substrate with W_18_O_49_ as efficient light absorbing material. And the porosity enables the melamine foam a high loading capability of W_18_O_49_. The final seawater desalination composite was fabricated by calcining the melamine foam loaded with W_18_O_49_ in N_2_ at 500 °C. The composite is beneficial for solar steam generation due to its capability of self‐floating at the water surface and good flexibility.

## 2 Characterization

The X‐ray diffraction (XRD) patterns were recorded by PANalytical B.V. Empyrean X‐ray Powder Diffraction with Cu Kα radiation over a range of 10–70° (2θ) with 0.02° per step. Scanning electron microscope (SEM) images were performed with JSM‐6700F electron microscope. X‐ray photoemission spectroscopy (XPS, Thermo ESCALAB 250) was performed using monochromatic Al Kα radiation (1486.6 eV). Solid UV–vis absorption was performed by using Lambda 950 spectroscope. The thermal image was taken by Fluke thermal imaging camera.

## 3 Results and Discussion

**Figure 1:**
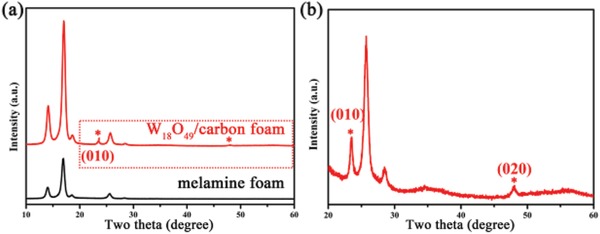
a) The XRD pattern of melamine foam and W_18_O_49_/carbon foam composite. b) The partial, enlarged XRD pattern of the selected area in (a).

**Figure** **1** shows the XRD patterns of melamine foam and W_18_O_49_/carbon foam composite. These two XRD patterns almost have the same diffraction peaks except from the additional peaks at ≈23° and 48° which could be clearly observed in Figure 1b (the partial magnified image of selected area in Figure 1a). This result reveals that the foam does not change into graphite carbon during the carbonization process under this temperature. And these additional peaks match well with the paralleled crystal faces of monoclinic W_18_O_49_ (*p2/m*, JCPDS No. 71‐2450),^24^ namely (010) and (020) faces, which illustrates that W_18_O_49_ is successfully loaded into the carbon foam. Furthermore, monoclinic W_18_O_49_ crystal could withstand lots of oxygen vacancies due to its unique crystal structure. And the oxygen vacancies can be tuned through the environment of redox reaction.^24^ Thus, this annealing process in N_2_ atmosphere does not change the original crystal phase.

The top and side view SEM images of the composite intuitively revealed that W_18_O_49_ is exactly caged into the carbon foam (W_18_O_49_ was dyed in red and the carbon foam was dyed in green). As a matter of fact, the most common morphology of W_18_O_49_ is nanowire due to its preferential growth direction along the 〈010〉 direction.^24^, ^34^, ^35^ While the W_18_O_49_ nanowires aggregate and interweave with the porous net of the foam during the annealing process, which exactly insures the retention of W_18_O_49_ in the porous foam during its practical application. This is important and essential for its practical and long‐term application.

**Figure 2:**
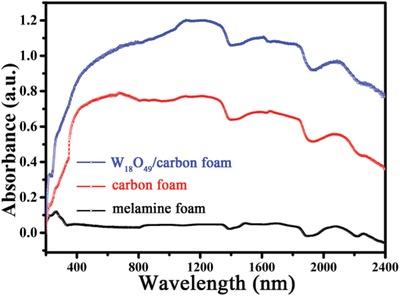
The UV–vis–NIR spectra of melamine foam, carbon foam, and W_18_O_49_/carbon foam, respectively.

**Figure 3:**
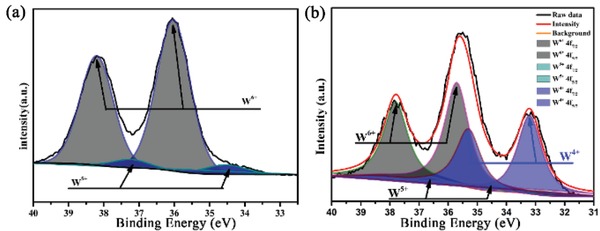
XPS spectra of W 4f orbital in W_18_O_49_ a) before and b) after being annealed in N_2_.

The melamine foam changes from white to black in color after the carbonization process. As a result, compared to the melamine foam, the absorbance of the carbon foam during the whole UV–vis–NIR spectrum increases dramatically as shown in **Figure** **2**. Whereas the W_18_O_49_/carbon foam exhibits the highest absorbance which could be attributed to two reasons. The first one is the porous net structure which increases the light scattering and the other one is the embedded strong solar absorption material. We have reported a photothermal conversion material due to its reduced tungsten (W^5+^ or W^4+^), which directly affect its photothermal conversion efficiency at a large extent.^24^ The most applied method to increase the amount of reduced tungsten (W^5+^ or W^4+^) is being annealed in inert or reductive atmosphere.^25^, ^36^ Thus, the annealing process of melamine foam and W_18_O_49_ in N_2_ not only helps the aggregation of W_18_O_49_ but also increases its light absorption. As shown in **Figure** **3**, after being annealed in N_2_ atmosphere, the total amount of reduced tungsten (W^5+^ or W^4+^) increase from 16.7% to 45% (calculated by its integral area), which could dramatically increase its solar absorption. And this is consistent with the UV–vis–NIR absorption result.

**Figure 4:**
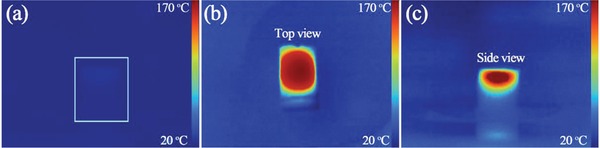
The thermal image of the W_18_O_49_/carbon foam composite a) before and b,c) after being irradiated by 808 nm laser.

**Figure 5:**
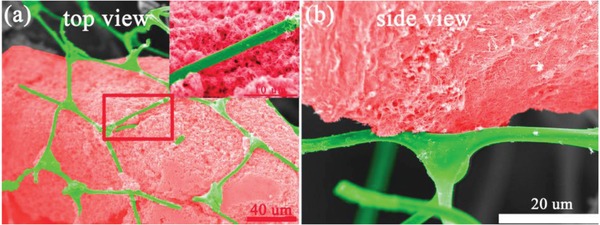
a) The top‐view SEM image of the W_18_O_49_/carbon foam composite; inset is the partial enlarged image of the selected area in red. b) The side‐view SEM image of the W_18_O_49_/carbon foam (W_18_O_49_ was dyed in red and the carbon foam was dyed in green).

Due to the large amount of reduced tungsten (W^5+^ or W^4+^), the W_18_O_49_/carbon foam composite should have an excellent photothermal conversion performance. **Figure** **4** shows the thermal image of the W_18_O_49_/carbon foam after being irradiated by a 808 nm laser. The top view thermal image (irradiated from top) illustrates that the heat is evenly distributed throughout the horizontal plane and the side view thermal image (irradiated from side) reveals that the heat is concentrated at the top of vertical plane. That is to say, the bottom carbon layer could reduce the heat loss by thermal diffusion which is unnecessary for evaporation. The side view thermal image also reveals a better photothermal conversion performance of W_18_O_49_ than that of the carbon foam because the up temperature is higher than the down temperature when they are irradiated at the same condition. Moreover, it also reveals that the W_18_O_49_ is just deposited on the surface of the carbon foam which is consistent with the side view SEM image (**Figure** **5**b). The self‐floating property of the composite benefits from the hydrophobic property of the carbon foam. While this could be simply realized by the carbonization process unlike the complicated surface modification to keep the product floating on the water surface.^37^ Based on the above discussion, the as‐prepared W_18_O_49_/carbon foam might have a practical application for efficient water evaporation under solar illumination due to its porous net structure and its capability of concentrating the heat at the surface of the foam.

**Figure 6:**
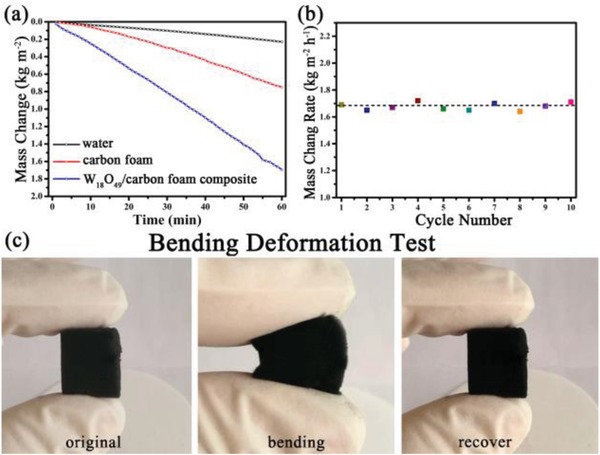
a) The mass of the evaporated water as a function of the time under 500 W Xe lamp irradiation. b) The cyclic performance of W_18_O_49_/carbon foam composite. c) The bending deformation test of W_18_O_49_/carbon foam composite.

**Figure** **6**a shows the mass changes of pure water, water with carbon foam, and water with W_18_O_49_/carbon foam composite under 500 W Xe lamp irradiation. The mass of evaporated water for these three different cases almost has a linear relationship with the time. The evaporation rates are 0.229, 0.751, and 1.694 kg m^−2^ h^−1^ for pure water, water with carbon foam, and water with W_18_O_49_/carbon foam composite, respectively. Apparently, the carbon foam loaded with W_18_O_49_ dramatically improves the water evaporation rate. The broadband absorption of carbon foam indeed increases the water evaporation rate which is 2.9 times higher than that of pure water. While the loading of a better photothermal conversion material W_18_O_49_ could further improve the evaporation rate up to 6.6 times. This water evaporation rate is much higher than that of the reported black TiO_2−_ *_x_* which is just 1.3 times of pure water.^37^ The cyclic performance is shown in figure 6b, the evaporation efficiency of W_18_O_49_/carbon foam almost keeps the same after ten cycles irradiation. Furthermore, its flexibility is evaluated by the bending deformation test as shown in Figure 6c. The W_18_O_49_ loaded carbon foam could recover its original shape immediately after releasing the bending stress which illustrates its excellent flexibility.

## 4 Conclusion

In summary, we have successfully fabricated a self‐floating W_18_O_49_/carbon foam composite which can be applied in efficient water evaporation. After annealing the efficient photothermal conversion material W_18_O_49_ and melamine foam in inert atmosphere, the product gained its self‐floating property as well as a better photo‐thermal conversion capability. The hydrophobic porous nature of the carbon foam makes it floating on the surface of water and the W_18_O_49_ could concentrate the heat at the top of the foam, which is beneficial for heat exchange at the interface of water and air. The evaporation rate for this composite is calculated to be 6.6 times higher than that of pure water under a solar simulator irradiation (500 W Xe lamp). And its good cyclic stability and flexibility makes it a prospective material in solar energy conversion field.

## 5 Experimental Section

*Synthesis of W_18_O_49_*: W_18_O_49_ nanomaterials were prepared by using a solvothermal method.^24^, ^34^ The experimental process is as follows: WCl_6_ (100 mg) was dispersed in 1‐butanol (60 mL) with sonication for 3 min. The obtained blue solution was transferred into a 100 mL Teflon‐lined autoclave and kept at 200 °C for 24 h. The product was washed with water and ethanol four times by centrifugation and then dried in the vacuum overnight.

*Synthesis of W_18_O_49_/Carbon Foam*: 50 mg as‐prepared W_18_O_49_ nanomaterial was dispersed in 10 mL water by ultrasonic treatment to form a homogenous dispersion. The W_18_O_49_ dispersion was quickly loaded into the melamine foam by dipping into the dispersion. The composite of W_18_O_49_ and melamine foam was annealed in N_2_ to form the self‐floating W_18_O_49_/carbon foam composite. And the mass ratio of W_18_O_49_ to the carbon foam was about 1.25 (50 mg W_18_O_49_ to a 0.005 g cm^−3^ density of carbon foam with 2 × 2 × 2 cm^3^). The W_18_O_49_ was just loaded at the surface of the carbon foam and its loading thickness was about 0.5 cm.

*Solar Steam Generation*: The self‐floating composite was evenly floated at the surface of 100 mL water in the beaker which was coated with thermal insulation material. The beaker was steadily placed in an electronic balance which records the mass change of the whole system during the simulated solar light illumination. The solar irradiation was simulated by a solar simulator xenon lamp at a power of 500 W.

## Conflict of Interest

The authors declare no conflict of interest.
